# Structure-activity study of furyl aryloxazole fluorescent probes for the detection of singlet oxygen

**DOI:** 10.1371/journal.pone.0200006

**Published:** 2018-07-02

**Authors:** Renzo P. Zanocco, Roger Bresoli-Obach, Santi Nonell, Else Lemp, Antonio L. Zanocco

**Affiliations:** 1 Departamento de Química Orgánica y Fisicoquímica, Facultad de Ciencias Químicas y Farmacéuticas, Universidad de Chile, Santiago, Chile; 2 Institut Químic de Sarrià, Universitat Ramon Llull, Barcelona, Spain; Universidade de Sao Paulo Instituto de Quimica, BRAZIL

## Abstract

In this study, we report the synthesis and the photochemical behavior of a series of new "click-on" fluorescent probes designed to detect singlet oxygen. They include a highly fluorescent chemical structure, an aryloxazole ring, linked to a furan moiety operating as singlet oxygen trap. Their activity depends on both the structure of the aryloxazole fluorophore and the electron-donating and electron-accepting properties of the substituents attached to the C-5 of the furan ring. All probes are selectively oxidized by singlet oxygen to give a single fluorescent product in methanol and produce negligible amounts of singlet oxygen themselves by self-sensitization. The most promising dyad, (E)-2-(2-(5-methylfuran-2-yl)vinyl)naphtho[1,2-d]oxazole, **FN-6**, shows outstanding reactivity and sensitivity: it traps singlet oxygen with a rate constant (5,8 ± 0.1) x 10^7^ M^-1^ s^-1^ and its fluorescence increases by a factor of 500 upon reaction. Analysis of the dyads reactivity in terms of linear free energy relationships using the modified Swain and Lupton parameter F and the Fukui condensed function for the electrophilic attack, suggests that cycloaddition of singlet oxygen to the furan ring is partially concerted and possibly involves an exciplex with a "more open" structure than could be expected for a concerted cycloaddition.

## Introduction

Singlet oxygen, (O_2_(^1^Δ_g_), hereafter ^1^O_2_), the first electronic excited state of molecular oxygen, is a well-known reactive oxygen species (ROS), that can diffuse and oxidize several types of biomolecules such as proteins, nucleic acids and lipids constituent of cell membranes [1–8]. Due to this reactivity, ^1^O_2_ is recognized as an important ROS capable of promoting a large variety of cell responses [9–11]. On the other hand, ^1^O_2_ is one of the most central cytotoxic ROS produced by exposure of sensitizers to light in photodynamic therapy, PDT [12–15].

To understand in depth the mechanisms associated with cellular stimuli originated by the presence of ^1^O_2_ and/or to appropriately control the photooxidation process in PDT treatments, the detection and quantification of ^1^O_2_ is undoubtedly one the most relevant and critical factors. Common ^1^O_2_ detection methods include electron paramagnetic resonance [16–18], chemiluminescence [19–21] and fluorescence spectroscopy [22–29]. These methods are based on the observation of a signal produced by a probe attached to a ^1^O_2_ chemical acceptor that responds to the oxidation stage of the acceptor. Typical chemical acceptors are electron-rich dienes, naphthalenes, anthracenes and furans.

Furan derivatives such as 2,5-diphenyl-3,4-isobenzofuran (DPBF) or 2,5-dimethylfuran, have been widely used as a ^1^O_2_ scavengers since the 70s [30,31]. They are advantageous chemical traps of ^1^O_2_ because they can be quantified by using routine analytical techniques such as spectrophotometry or gas chromatography, they react with ^1^O_2_ mainly via a chemical channel to form endoperoxides with a minimal or null contribution of physical quenching [32], and the overall quenching rate constant, *k*_*q*_, shows a very modest solvent dependence. In addition, furans react specifically with ^1^O_2_ and thus are ideally suited to develop selective ^1^O_2_ probes [33,34].

Our group has long been interested in the detection and quantification of ^1^O_2_ using furan derivatives [27,35,36]. We recently started a program for the design, synthesis and study of new “click-on” furan-based fluorescent probes for ^1^O_2_ sensing [27]. The novel molecular entities are dyads comprising an aryloxazole fluorescent moiety linked to a furan trap. In their native state, the inherently strong fluorescence of the aryloxazole moiety is quenched by the electron-rich furan. Upon reaction with ^1^O_2_, the furan is oxidized and the quenching process ceases to operate, restoring the intrinsically strong fluorescence of the aryloxazole ring. Moreover, the absorption and fluorescence spectra of the native and oxidized forms of the probe are different, which allows the selective photoexcitation of either form, thereby further enhancing the fluorescence contrast to an unmatched level [22,27].

**Fig 1:**
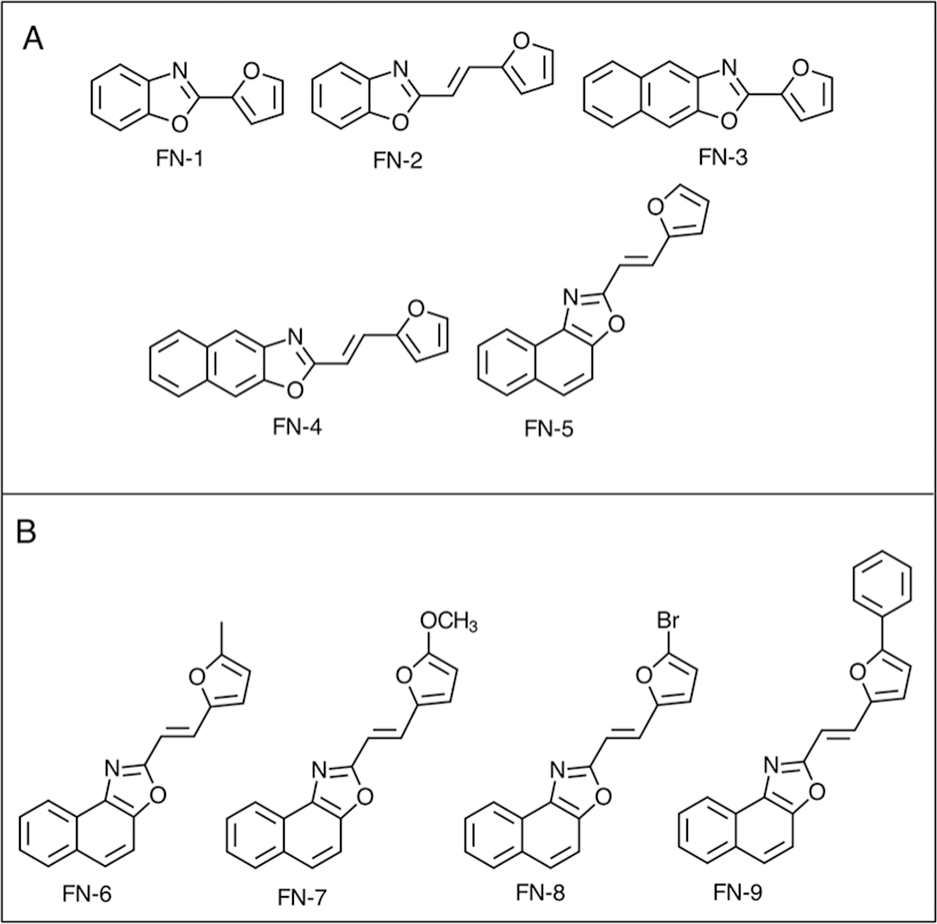
**A**. **Unsubstituted furyl- and furylvinyl aryloxazoles with different aryl moieties linked to the furan ring**. **FN-1**: 2-(furan-2-yl)benzo[1,2-d]oxazole, **FN-2**: (E)-2-(2-(furan-2-yl)vinyl)benzo[1,2-d]oxazole, **FN-3**: 2-(furan-2-yl)naphtho[2,3-d]oxazole, **FN-4**: (E)-2-(2-(furan-2-yl)vinyl)naphtho[2,3-d]oxazole, **FN-5**: (E)-2-(2-(furan-2-yl)vinyl)naphtho[1,2-d]oxazole. **B**. **Furyl vinyl naphthoxazoles with different substituents in the furan ring**. **FN-6**: (E)-2-(2-(5-methyl-furan-2-yl)vinyl)naphtho[1,2-d]oxazole, **FN-7**: (E)-2-(2-(5-methoxy-furan-2-yl)vinyl)naphtho[1,2-d]oxazole, **FN-8**: (E)-2-(2-(5-bromo-furan-2-yl)vinyl)naphtho[1,2-d]oxazole, **FN-9**: (E)-2-(2-(5-phenyl-2-furan-2-yl)vinyl)naphtho [1,2-d]oxazole.

Having established the proof of concept for this new family of probes, we have now set out to explore the effect of structural modifications on the performance of aryloxazole-furan probes and we report herein the results of our studies. We aimed at rationalising the fluorescent response of the probes and their reactivity towards ^1^O_2_ to understand the factors affecting their performance. Thus, we have been able to design an optimum candidate by modifying the structure of the aryloxazole moiety (Fig 1A) and by including different substituents at the C-5 position of the furan ring (Fig 1B).

## Materials and methods

### Materials

Perinaphthenone (Sigma-Aldrich), new methylene blue (NMB, Sigma-Aldrich), anthracene (Sigma-Aldrich), naphthalene (Sigma-Aldrich), 1-methyl-2-pyrrolidone, (Sigma-Aldrich), 2-methylbenzo[1,2-d]oxazole (Sigma-Aldrich), 2-methylnaphtho[1,2-d]oxazole (AK Scientific, Inc.), 2-methylnaphtho[3,2-d]oxazole (TCI), 2-aminophenol (Sigma-Aldrich), 1-amino-2-naphthol hydrochloride (Sigma-Aldrich), 2-amino-3-naphthol (Sigma-Aldrich), furfural (Sigma-Aldrich), 5-methylfurfural, (Sigma-Aldrich), 5-bromofurfural (Sigma-Aldrich), 5-phenylfurfural (Sigma-Aldrich) and furoyl chloride (Sigma-Aldrich) were used as received. All solvents used were UV or HPLC grade.

### Spectroscopic measurements

Absorption spectra were recorded on a UV4 spectrophotometer (Unicam). Fluorescence emission spectra were recorded on a Quattro III (OBB) or on a PC1 (ISS) spectrofluorometers. Fluorescence quantum yields (Φ_F_) were determined by the comparative method described by Eaton and Demas [37,38], using quinine sulphate in 0.1 N sulphuric acid (Φ_F_ = 0.55) or naphthalene in ethanol (Φ_F_ = 0.21) as references [39]. The absorbance of sample and reference solutions was set below 0.1 at the excitation wavelength and the fluorescence emission spectra were corrected using rhodamine G as reference. Sample quantum yields were evaluated using Eq (1):

$$\Phi_{X}=\left(\frac{Grad_{X}}{Grad_{Act}}\right)\left(\frac{\eta_{X}^{2}}{\eta_{Act}^{2}}\right)\Phi_{Act}$$ (1)

where *Grad*_*X*_ and *Grad*_*Act*_ are the slope of integrated fluorescence vs. absorbance plots for the sample and the actinometer, respectively, and *η*_*x*_ and *η*_*Act*_ are the refractive index of sample and actinometer solutions, respectively. All measurements were carried out in nitrogen-purged solutions at (20.0 ± 0.5)°C. Fluorescence decays were recorded with a time-correlated single photon counting system (Fluotime 200; PicoQuant GmbH, Berlin, Germany) equipped with a red-sensitive photomultiplier. Excitation was achieved by means of a 375 nm picosecond diode laser working at 10 MHz repetition rate. The counting frequency was maintained always below 1%. Fluorescence lifetimes were analyzed using the PicoQuant FluoFit 4.0 software.

Light irradiation of NMB at 660 ± 5 nm in steady-state experiments, was performed in 1 cm spectrophotometer cuvettes using a ThorLabs M660L4 LED light source (15 mW cm^-2^). The same setup was employed to determine the chemical reaction rate constant, *k*_*r*_, in methanol. The distance between the light source and the cell was set for each experiment so that the initial substrate concentration would diminish about 50% in 15 min. Dyad consumption was evaluated by observing the decrease of absorbance over time and *k*_r_ values were derived from the slopes of the linear pseudo first-order photoconsumption plots using 9,10-dimethylanthracene (*k*_*r*_ = 6,3 x 10^7^ M^-1^ s^-1^ [40]) as reference. Photooxidation products for HPLC-MS analysis were prepared by long-term irradiation of the samples (> 90% conversion).

### Singlet oxygen measurements

The phosphorescence of ^1^O_2_ was detected by means of a customized PicoQuant Fluotime 200 system. A Diode-pumped Q-switched laser (Pulselas-A-660-50, AlphaLas) working at 2 kHz repetition rate was used for excitation of NMB at 660 nm. The luminescence exiting from the side of the sample was filtered by a 1100 nm cut-off filter (Edmund Optics) and a narrow bandpass filter at 1270 nm (NB-1270-010, Spectrogon) to remove any scattered laser radiation. A plane-convex lens (23 mm diam. X 75 mm) was used to focus the light emitted onto the photomultiplier window. A near-IR sensitive photomultiplier tube assembly (H1033A-45; Hamamatsu Photonics) was used as detector. Photon counting was achieved with a multichannel scaler (PicoQuant’s Nanoharp 250). Time-resolved emission signals *S*_*t*_ were analyzed using the PicoQuant FluoFit 4.0 data analysis software to extract lifetime (*τ*_*t*_ and *τ*_Δ_) and amplitude (S_0_) values. Quantum yields for ^1^O_2_ production (Φ_Δ_) were calculated from the amplitudes using the following Eqs (2)–(4):

$$S_{t}=S_{0}\left(\frac{\tau_{\Delta }}{\tau_{\Delta }-\tau_{t}}\right)\left(e^{\frac{\tau_{t}}{\tau_{\Delta }}}-e^{\frac{\tau }{\tau_{t}}}\right)$$ (2)

$$S_{0}\propto \Phi_{\Delta }$$ (3)

$$\Phi_{\Delta }\left(sample\right)=\Phi_{\Delta }\left(ref\right)\left(\frac{S_{0}\left(sample\right)}{S_{0}\left(ref\right)}\right)$$ (4)

Perinaphthenone was used as reference for which Φ_Δ_ = 1 was taken [41].

The rate constant for ^1^O_2_ quenching by the dyads (*k*_*q*_) was determined by measuring the ^1^O_2_ lifetime as a function of the dyad concentration. ^1^O_2_ was generated by photoexcitation of a 50 μM NMB solution at 665 nm and the concentration of the dyads was varied in the range (0.1–1 mM). A plot of the reciprocal lifetime, 1/*τ*_Δ_, vs. the concentration of the dyad afforded *k*_*q*_ as the slope of the linear fit, Eq (5),

$$\frac{1}{\tau_{\Delta }}=\frac{1}{\tau_{\Delta }^{0}}+k_{q}\left[Dyad\right]$$ (5)

where τ^0^_Δ_ is the ^1^O_2_ lifetime in the neat solvent.

### Synthesis of furyl aryloxazoles

Furyl derivatives of aryloxazoles were synthesized using the method of El’chaninov et al [42]. Typically, 1.1 mmol of amino arylalcohol and 1 mmol of furoyl chloride in 4 mL of dry 1-methyl-2-pyrrolidone were stirred under nitrogen by 1 h. Addition of 6 mL of cold water gives a precipitate that was filtered and washed with 20 mL of cold acetonitrile. Recrystallization of the solid in acetonitrile affords the product with high purity.

#### 2-(furan-2-yl)benzo[1,2-d]oxazole, FN-1

^1^H-NMR (400 MHz, DMSO-d_6_); δ: 8.06 (dd, 1H, J = 1.8; 0.8 Hz, Fu-H_1_), 7.75 (m, 1H, Ar-H_6_, Ar-H_7_), 7.45 (dd, 1H, J = 3.5; 0.8 Hz, Fu-H_2_), 7.41 (m, 1H, Ar-H_4_, Ar-H_5_), 6.80 (dd, 1H, J = 3.5; 1.8 Hz, Fu-H_3_); ^13^C-NMR (100 MHz, DMSO-d_6_); δ: 155.07; 149.98; 147.58; 142.06; 141.56; 126.04; 125.53; 120.22; 115.58; 113.25; 111.32; IR(KBr) υ(cm^-1^): 3420, 3108, 1634, 1450, 1014, 955; Elem. Anal.: exp. C 73.97%, H 4.18%, N 6.75%; calc. C 71.35%, H 3.81%, N 7.56%; MS(ESI) *m/z*: 186.05.

#### 2-(furan-2-yl)naphtho[2,3-d]oxazole, FN-3

^1^H-NMR (400 MHz, DMSO-d_6_) δ: 8.29 (s, 1H, Ar-H_4_), 8.22 (s, 1H, Ar-H_5_), 8.14 (dd, 1H, J = 1.7; 0.8 Hz, Fu-H_1_), 8.06(m, 2H, Ar-H_7_), 7.58 (dd, 1H, J = 3.6, 0.8 Hz, Fu-H_2_), 7.51 (m, 2H, Ar-H_6_), 6.85 (dd, 1H, J = 3.6; 1.7 Hz, Fu-H_3_), ^13^C-NMR (100 MHz, DMSO-d_6_) δ: 156.83, 148.87; 148.29; 141.83; 141.50; 131.82; 131.69; 128.73; 128.36; 126.06; 125.38; 117.41; 116.99; 113.50; 106.95, IR(KBr) υ(cm^-1^): 3418, 3051, 1652, 1305, 1080, 1011, Elem. Anal.: exp.: 76.18%, H 4.12%, N 6.16% calc. C 76.59%, H 3.68%, N 6.95%; MS (ESI+) *m/z* 235,90.

### Synthesis of furyl vinyl aryloxazoles

Furyl vinyl aryloxazoles were obtained using the method of Zajac et al. **[43]**, employing dimethylsulfoxide as solvent and KOH as the base. Typically 1.1 mmol of 2-methylaryloxazole and 1 mmol of furfural were dissolved in 2 mL of dimethylsulfoxide. Then, were added 0.124 mL of an aqueous solution of KOH 50% and the mixture was stirred by 1 h at room temperature. Addition of 10 mL of water afforded a yellow precipitate, which was filtered, washed with cold water and cold methanol and recrystallized from acetonitrile giving the product in high purity with yields between 50–60%.

#### (E)-2-(2-(furan-2-yl)vinyl)benzo[1,2-d]oxazole, FN-2

^1^H-NMR (400 MHz, DMSO-d_6_); δ: 7.85 (dd, 1H, J = 1.8; 0.5 Hz, Fu-H1), 7.68 (m, 1H, Ar-H7), 7.62 (d, 1H, J = 16.1 Hz, Ar-H5), 7.36 (m, 2H, Ar-H6), 6.95 (dd, 1H, J = 3.4; 0.5 Hz, Fu-H2), 6.87 (d, 1H, J = 16.1 Hz, Vi-H4), 6.65 (dd, 1H, J = 3.4; 1.8 Hz, Fu-H3); ^13^C-NMR (100 MHz, DMSO-d_6_); δ: 162.53; 151.27; 150.23; 146.02; 142.21; 126.81; 125.81; 125.19; 119.93; 115.20; 113.29; 111.01; 110.96; IR (KBr) υ (cm^-1^); 3429, 3131, 2928, 1636, 1452, 1085, 942; Elem. Anal.: exp. C 71.11%, H 3.87%, N 7.32%; calc. C 73.92%, H 4.29%, N 6.63%; MS (ESI+) *m/z*: 212.21.

#### (E)-2-(2-(furan-2-yl)vinyl)naphtho[2,3-d]oxazole, FN-4

^1^H-NMR (400 MHz, DMSO-d_6_) δ: 8.23 (s, 1H, Ar-H7), 8.14 (s, 1H, Ar-H6), 8.05 (m, 2H, Ar-H9), 7.90 (dd,1H, J = 1.7; 0.4 Hz, Fu-H1), 7.75 (d, 1H, J = 16.1 Hz, Vi-H4), 7.50 (m, 2H, Ar-H8), 7.03 (dd, 1H, J = 3.4; 0.5 Hz, Fu-H3), 6.93 (d, 1H, J = 16.1 Hz, Vi-H6), 6.68 (dd, 1H, J = 3.4; 1.8 Hz, Fu-H2), ^13^C-NMR (100 MHz, DMSO-d_6_) δ: 164.60; 151.25; 149.16; 146.46; 142.24; 131.84; 131.57; 128.73; 128.31; 128.22; 125.97; 125.21; 117.07; 116.03; 113.47; 110.80; 106.45, IR(KBr) υ(cm^-1^) 3432, 3052, 2926, 1646, 1248, 1017, Elem. Anal.: exp. C 77.98%, H 4.18%, N 5.41%; calc. C 78.15%, H 4.24%, N 5.36%; MS (ESI+) *m/z* 261.82.

#### (E)-2-(2-(furan-2-yl)vinyl)naphtho[1,2-d]oxazole, FN-5

^1^H-NMR (400 MHz, DMSO-d_6_) δ: 8.36 (d, 1H, J = 8.3 Hz, Ar-H8), 8.09 (d, 1H, J = 8.2 Hz, Ar-H11), 7.95 (d, 1H, J = 8.9 Hz, Ar-H7), 7.89 (d, 1H, J = 8.9 Hz, Ar-H6), 7.87 (d, 1H, J = 1.7 Hz, Fu-H1), 7.70 (ddd, 1H, J = 8.2; 6.9; 1.2 Hz, Ar-H9), 7.66 (d, 1H, J = 16.1 Hz, Vi-H6), 7.59 (ddd, 1H, J = 8.2; 6.9; 1.3 Hz, Ar-H10), 6.99 (d, 1H, J = 16.2 Hz, Vi-H4), 6.97 (d, 1H, J = 3.3 Hz, Fu-H3), 6.66 (dd, 1H, J = 3.4; 1.8 Hz, Fu-H2); ^13^C-NMR (100 MHz, DMSO-d_6_) δ: 162.09; 151.43; 147.67; 145.88; 137.44; 131.30; 129.30; 127.77; 126.79; 126.04; 125.94; 125.90; 121.96; 114.92; 113.29; 111.44; 111.13; IR (KBr) υ(cm^-1^); 3435, 3059, 2925, 1633, 1463, 1018; MS (ESI+) *m/z* 261.88.

#### (E)-2-(2-(5-methyl-furan-2-yl)vinyl)naphtho[1,2-d]oxazole, FN-6

^1^H-NMR (400 MHz, DMSO-d_6_) δ: 8.35 (d, 1H, J = 8.3 Hz, Ar-H8), 8.08 (d, 1H, J = 8.2 Hz, Ar-H11), 7.93 (d, 1H, J = 8.8 Hz, Ar-H7), 7.88 (d, 1H, J = 8.9 Hz, Ar-H6), 7.69 (ddd, 1H, J = 8.2; 6.9, 1.2 Hz, Ar-H10), 7.58 (d, 1H, J = 16.3 Hz, Vi-H5), 7.57 (ddd, 1H, J = 8.3; 6.9; 1.2 Hz, Ar-H9), 6.85 (d, 1H, J = 16.1 Hz, Vi-H4), 6.85 (dd, J = 3.3; 0.5 Hz, Fu-H2), 6.29 (dd, J = 3.3; 1.0 Hz, Fu-H3), 2.37 (s, 3H, C-H1); ^13^C-NMR (100 MHz, DMSO-d_6_) δ: 162.37; 155.42; 150.09; 147.59; 137.48; 131.28; 131.05; 129.28; 127.69; 126.54; 125.97; 125.89; 121.97; 116.58; 111.39; 109.83; 109.40; 114.11; IR (KBr) υ(cm^-1^); 3428, 3056, 2919, 1630, 1483, 1020; Elem. Anal.: exp. C 77.012%, H 4.63%, N 4.99%; calc. C 78.53%, H 4.76%, N 5.09%; MS (ESI+) *m/z* 276.17.

#### (E)-2-(2-(5-bromo-furan-2-yl)vinyl)naphtho[1,2-d]oxazole; FN-8

^1^H-NMR (400 MHz, DMSO-d_6_) δ: 8.36 (d,1H, J = 8.3 Hz, Ar-H7), 8.09 (d, 1H, J = 8.2 Hz, Ar-H10), 7.95 (d, 1H, J = 8.9 Hz, Ar-H6), 7.89 (d, 1H, J = 8.9 Hz, Ar-H5), 7.70 (ddd, 1H, J = 8.2; 6.9; 1.2 Hz, Ar-H8), 7.60 (d, 1H, J = 16.1 Hz, Vi-H3), 7.59 (ddd, 1H, J = 8.2; 6.9; 1.3 Hz, Ar-H9), 7.00 (d, 1H, J = 3.0 Hz, Fu-H2), 6.98 (d, 1H, J = 16.3 Hz, Vi-H4), 6.79 (d, 1H, J = 3.5 Hz, Fu-H1); ^13^C-NMR (100 MHz, DMSO-d_6_) δ: 199.78; 161.83; 153.62; 147.73; 131.31; 129.32; 127.82; 126.96; 126.09; 125.92; 124.71; 121.6; 117.08; 115.40; 114.53; 111.72; 111.45; IR (KBr) υ(cm^-1^); 3432, 3061, 1632, 1460, 1085, 1006; Elem. Anal. exp. C 60.22%, H 3.15%, 4.05%; calc. C 61.04%, H 3.41%, N 3.95%; MS (ESI+) *m/z* 339.89.

#### (E)-2-(2-(5-phenyl-2-furan-2-yl)vinyl)naphtho[1,2-d]oxazole, FN-9

^13^C-NMR (100 MHz, DMSO-d_6_) δ: 162.30; 155.33; 151.16; 147.69; 137.55; 131.31; 129.47; 129.45; 129.40; 129.32; 128.87; 127.77; 126.75; 125.93; 125.41; 124.56; 124.46; 124.43; 121.96; 117.42; 111.44; 111.13; 109.39; IR(KBr) υ(cm^-1^); 3430, 3036, 1628, 1470, 1027, 1006; Elem. Anal.: exp. C 82.36%, H 4.59%, N 4.10%, calc. C 82.3%, H 4.88%, N 3.99%.

## Results and discussion

### Photophysical characterization of unsubstituted furyl aryloxazoles

**Fig 2:**
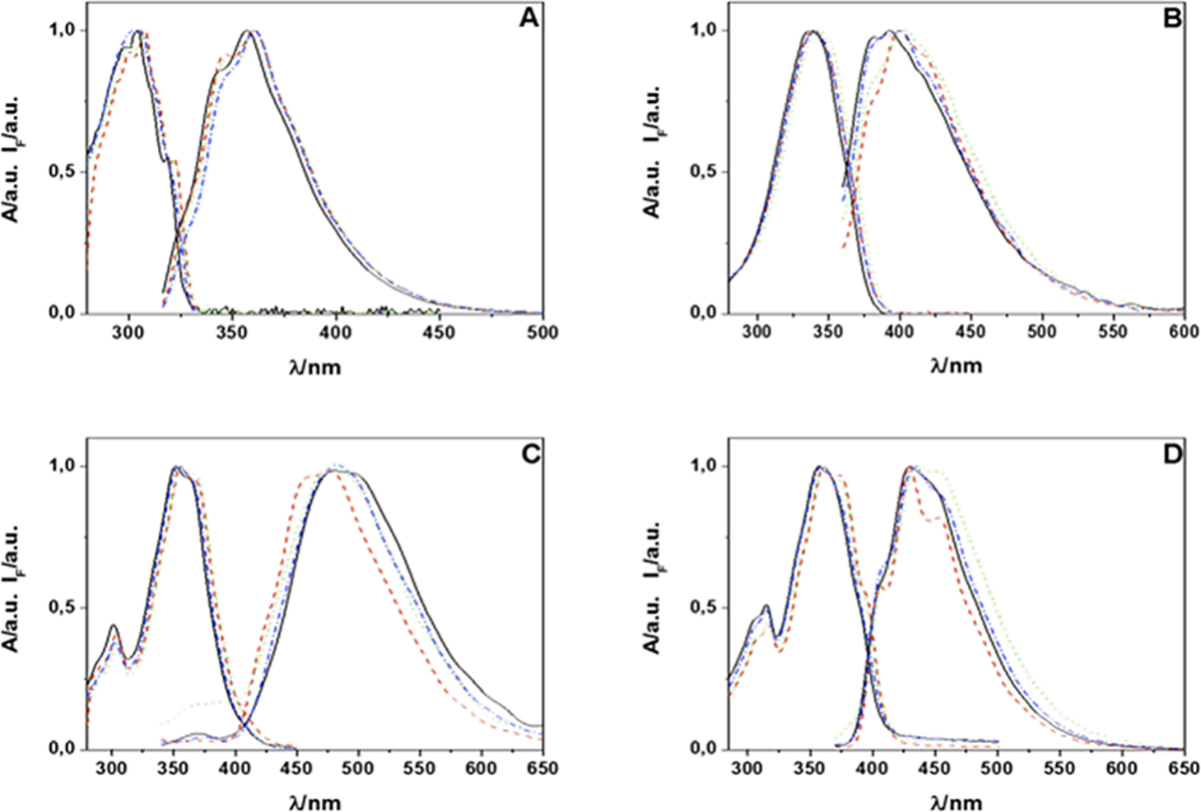
Absorption and emission spectra of probes FN-1 (A), FN-2 (B), FN-4 (C) and FN-5 (D) in solvents representatives of the polarity scale, acetonitrile (solid, black line), benzene (dash red line), dimethylformamide (dot green line), methanol (dash-dot light blue line).

Compounds **FN-1** to **FN-5** are dyads composed by an unsubstituted furan ring linked to an aryloxazole moiety through either a single C-C bond or a vinyl bridge. The aim of studying this series of compounds was to determine the most promising aryloxazole and link structures to detect and quantify ^1^O_2_. Absorption spectra of dyads **FN-1**, **FN-2**, **FN-4** and **FN-5** in solvents representative of the polarity scale are shown in Fig 2.

As can be observed in Fig 2 and from the data in Table 1, the position of lowest-energy band is insensitive to the solvent polarity, a behavior previously observed for related compounds [27,44]. Furthermore, the large value of the experimental molar absorption coefficients and molecular-orbital analysis of the minimum energy structures obtained from DFT calculations (6311g+dp orbital base) with Gaussian 04W indicate that the lowest energy bands correspond to π-π* transitions. Also, comparison of wavelength maxima of **FN-2** (naphthalene-like), **FN-4** (anthracene-like) and **FN-5** (phenanthrene-like), shows that the greater aromaticity of the dyad the smaller the transition energy [45–47].

**Table 1:** Absorption maxima and molar absorption coefficient of furyl aryloxazoles in representative solvents of different polarity.

Solvent	λ_max_/nm (ε/M^-1^ cm^-1^)				
	FN-1	FN-2	FN-3	FN-4	FN-5
**ACN**	304(28472)	354(29712)	332(35587)	334(53381)	357(32500)
**Benzene**	307(29166)	356(23613)	337(34665)	340(47996)	362(20000)
**DMF**	307(30753)	356(27230)	337(37468)	339(51523)	362(19722)
**Methanol**	307(35138)	356(30064)	337(37058)	340(53992)	360(27500)

On the other hand, these results indicate a strong electronic coupling between the oxazole moiety and the furan ring as visualized from the shape of the HOMO orbitals for **FN-1**, **FN-4** and **FN-5** (S1 Fig). The spectra of 2-(2-(furan-2-yl)ethyl)naphtho[1,2-d]oxazole, compound that has a saturated bridge between the aryloxazole group and the furan ring, shows a 40 nm blue-shifted absorption maximum [27].

The fluorescence spectra dyads in the same solvent set show a similar trend (Fig 2) with the position of the emission maxima nearly independent of the solvent polarity. Fluorescence quantum yields, measured using the Eaton and Demas method [37,38], are included in Table 2. For comparison, aryloxazoles typically show fluorescence quantum yields in the range of 0.7 to 1 [44,48,49] when aromatic substituents, different of furyl, are linked to the vinyl bridge, e.g. (E)-2-(4-methylstyryl)naphtho[1,2-d]oxazole [27], or in (E)-4-(2-(naphtho[1,2-d]oxazole-2-yl)vinyl)benzonitrile [44].

**Table 2:** Fluorescence quantum yields for unsubstituted furyl- and furyl vinyl aryloxazoles.

Solvent	Ф_F_				
	FN-1	FN-2	FN-3	FN-4	FN-5
**ACN**	0,20	< 0.01	0,43	0,05	0,02
**Benzene**	0,30	< 0.01	0,73	0,08	0,09
**DMF**	0,26	< 0.01	0,46	0,04	0,02
**Methanol**	0,17	< 0.01	0,33	0,03	0,09

The furyl derivatives clearly behave in a different way. Furyl vinyl aryloxazoles, **FN-2**, **FN-4** and **FN-5**, show a very low fluorescence quantum yield that, in addition, is essentially independent of the solvent polarity, i.e. when the furyl group is linked to the aryloxazole moiety through a vinyl bridge, the intrinsic fluorescence of aryloxazole is quenched by the furyl substituent [27]. This is a very important piece of information because the biological cell contains microenvironments of very different polarity. On the contrary, when the furyl substituent is linked directly to the oxazole ring, e.g., in **FN-1** or **FN-3**, the intramolecular quenching process does not operate and the fluorescence quantum yields are in the range of 0.2 to 0.7.

### Reactivity of unsubstituted furyl aryloxazoles towards singlet oxygen

To evaluate the reactivity of the probes with ^1^O_2_ we employed steady-state experiments to observe the evolution of absorption and emission spectra, and time resolved methods to measure changes in the decay kinetics of ^1^O_2_ luminescence.

**Fig 3:**
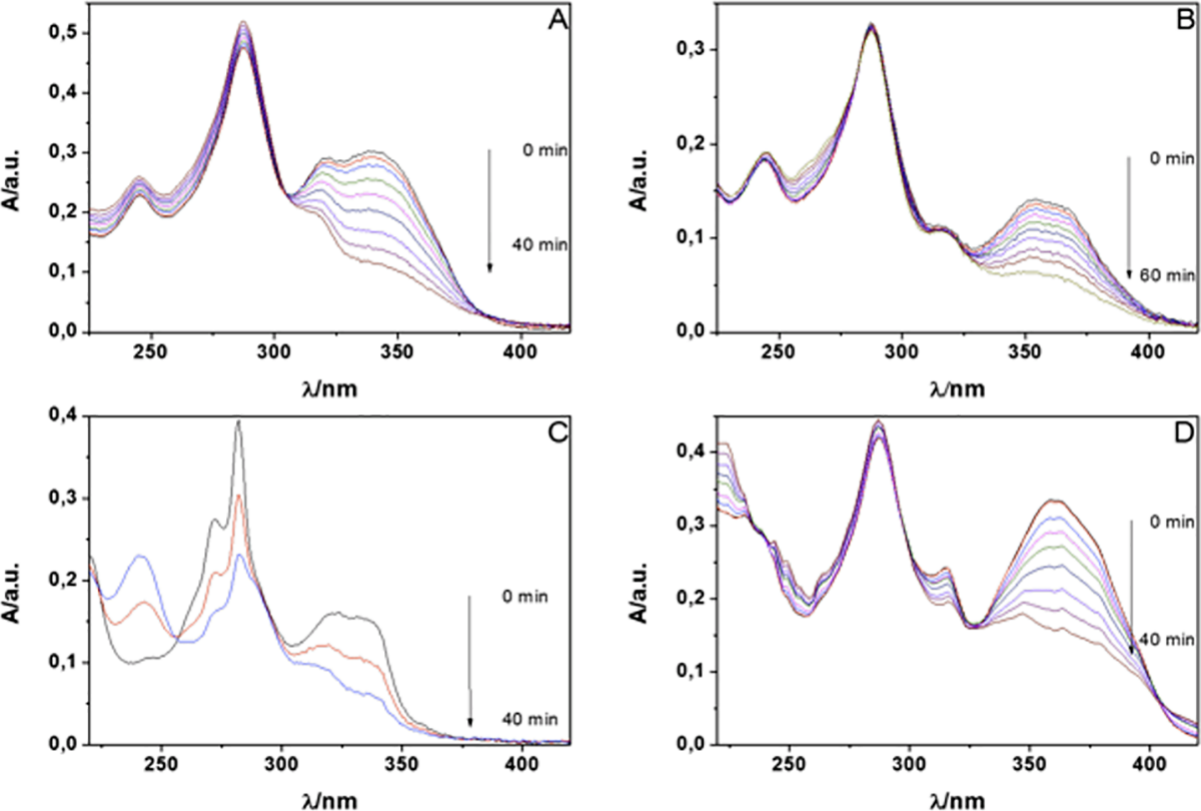
Evolution of the lower energy absorption band of dyads FN-2 (A), FN-3 (C), FN-4(B) y FN-5(D), due to reaction with ^1^O_2_ produced by photosensitization. Solvent: methanol; Sensitizer: NMB; Irradiation: Led, 660 nm.

Fig 3, shows the evolution of absorption spectra of **FN-2**—**FN-5**, observed over the course of 40 min irradiation of a solution in the presence of NMB as sensitizer.

All probes showed a strong absorbance decrease of the lowest energy band in a light-dose dependent fashion, a result that indicates that reaction occurs between ^1^O_2_ and the dyad. A careful examination of the spectra corresponding to **FN-2**, **FN-4** and **FN-5**, also reveal clear isosbestic points at 305, 289 and 298 nm, respectively, suggesting that a mayor photoproduct is formed when a furyl vinyl aryloxazole is the substrate. HPLC experiments confirm that only one product is formed in the photooxidation reaction (S2 Fig).

**Fig 4:**
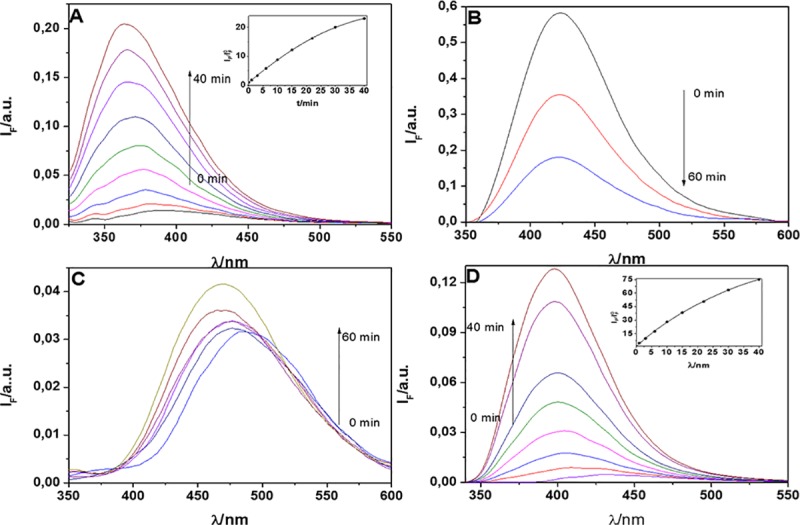
Changes in the fluorescence spectra of dyads FN-2 (A), FN-3 (B), FN-4(C) y FN-5(D), due to reaction with ^1^O_2_ produced by photosensitization. Solvent: methanol; Sensitizer: NMB; Irradiation: Led, 660 nm, irradiation time: 40 min. Inset: Increase of relative fluorescence with the irradiation time.

The modification of the absorption spectrum upon oxidation is a distinctive feature of this family of probes as compared to the most popular ones. The main benefit is that it allows to selectively exciting the fluorescence of the oxidized probe, which leads to very high fluorescent enhancement. The fluorescence changes upon reaction with ^1^O_2_ are shown in Fig 4.

**FN-2** and **FN-5** show an important fluorescence increase as photooxidation proceeds, 25- and 70-fold, respectively, exciting at their optimum wavelength (290 nm and 330 nm, respectively). In contrast, **FN-4** shows only minor changes and **FN-3**, on the other hand, shows a 3-fold decrease in fluorescence. The total (physical and reactive) ^1^O_2_ quenching rate constant (*k*_*q*_) for **FN-2** and **FN-5** were measured by observing singlet oxygen luminescence decay in time-resolved experiments. Lineal Stern-Volmer plots were obtained from which *k*_*q*_ values equal to (1,37 ± 0,06) x 10^7^ M^-1^ s^-1^ and (1,16 ± 0,04) x 10^7^ M^-1^ s^-1^ in ACN and (0,35 ± 0,02) x 10^7^ M^-1^ s^-1^ and (0,27 ± 0,02) x 10^7^ M^-1^ s^-1^ in methanol, respectively, were determined. Notice that the *k*_*q*_ values in methanol, are 25- and 36-fold lower, respectively, than that of 2-methylfuran in the same solvent (*k*_*q*_ = 9,9 x 10^7^ M^-1^ s^-1^) [34]. This important decrease in the furan reactivity is consistent with a substantial electronic delocalization of the furan π-electrons through the vinyl bridge.

Data reported in this section indicate that the most promising dyads for ^1^O_2_-detection are **FN-2** and **FN-5**, in which the furan and aryloxazole rings are linked by a vinyl bridge. Although **FN-5** is slightly less reactive than **FN-2**, it shows a larger fluorescence enhancement. We therefore selected **FN-5** as a basis for further development of fluorogenic ^1^O_2_ probes and proceeded to evaluate the effect of substituents in C-5 of the furan ring.

### Photophysical characterization of substituted furyl vinyl naphthoxazoles

**Fig 5:**
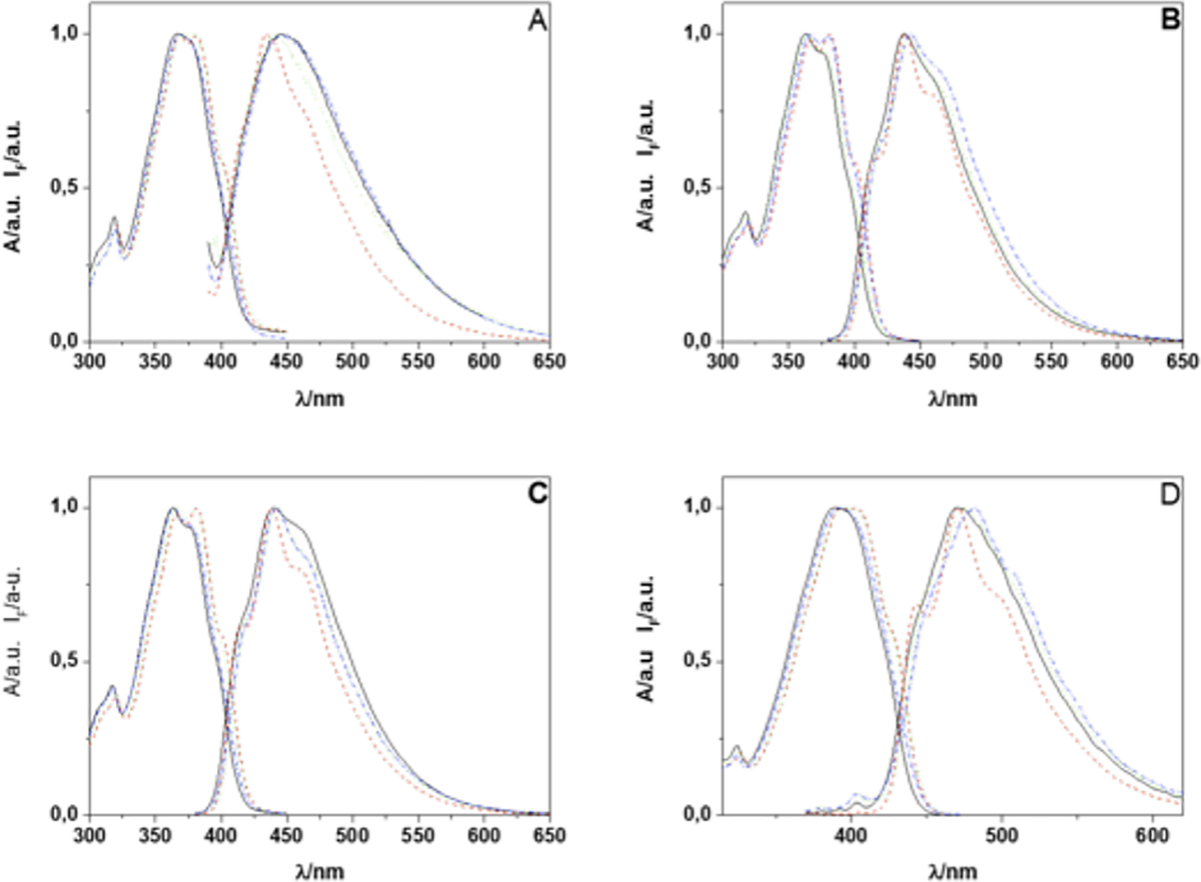
Absorption and emission spectra of probes FN-6 (A), FN-7 (B), FN-8 (C) and FN-9 (D) in solvents representatives of the polarity scale: acetonitrile (solid, black line), benzene (dash red line), dimethylformamide (dot green line), methanol (dash-dot light blue line).

Compounds **FN-6** to **FN-9** (Fig 1B) are dyads related to **FN-5** that include electron-withdrawing and electron-donating groups in position C-5 of the furyl moiety. Their absorption spectra are shown in Fig 5. As for compounds belonging to the first series, they are nearly independent on the solvent polarity, the absorption coefficients are in the range 25,000–50,000 M^-1^ cm^-1^, and molecular calculation analysis showed that the lowest energy transition is also π,π*. It is worth mentioning that the λ_max_ value is red-shifted by 30 nm for **FN-9**.

Likewise, their emission spectra (Fig 5) are also independent of the solvent polarity. Emission quantum yields are very low, particularly in polar solvents such as methanol, consistent with intramolecular charge transfer quenching the fluorescence emission [27]. Fluorescence lifetimes, Table 3, are around of 6 ns, independent of solvent and similar to those measured for other naphthoxazole derivatives [44].

**Table 3:** Fluorescence quantum yields for substituted furyl vinyl naphthoxazoles. In parenthesis fluorescence lifetime in ns.

Solvent	Ф_F_ (τ/ns)			
	FN-6	FN-7	FN-8	FN-9
**ACN**	0.004 (4.6)	0.014 (5.9)	0.014 (6.5)	0.014 (4.3)
**Benzene**	0.025 (6.3)	0.083 (5.9)	0.088 (6.8)	0.109 (7.2)
**DMF**	0.007 (5.6)	0.020 (5.9)	0.021 (6.4)	0.029 (6.8)
**Methanol**	0.003 (6.0)	0.008 (6.2)	0.010 (6.3)	0.010 (6.0)

### Reactivity of substituted furyl vinyl naphthoxazoles towards singlet oxygen

**Fig 6:**
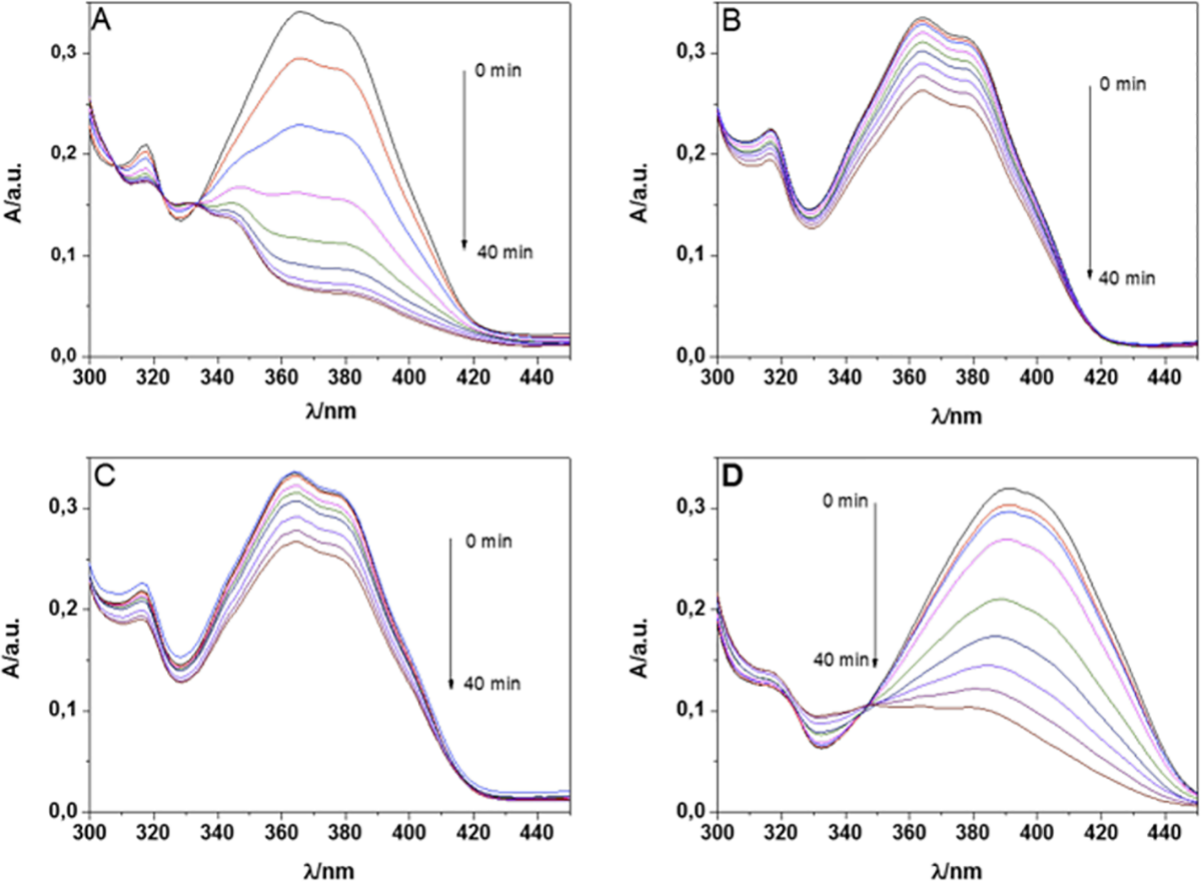
Absorption spectrum changes for FN-6 (A), FN-7 (B), FN-8 (C) and FN-9 (D) upon reaction with ^1^O_2_ generated by irradiation of NMB in methanol.

The ability of probes **FN-6 –FN-9** to react with ^1^O_2_ was evaluated using steady-state and time-resolved methods. ^1^O_2_ was photogenerated by irradiation of the sensitizer NMB in methanol. Changes in the absorption spectra of the probes upon reaction with ^1^O_2_ are shown in Fig 6. All dyads showed a notable loss of absorbance upon reaction with ^1^O_2_ and the relative rates of photobleaching were **FN-6** ≈ **FN-9** > **FN-5** >**FN-7** ≈ **FN-8**. Clear isosbestic points were observed, indicating a clean reaction with a single major product, which was confirmed by HPLC.

**Fig 7:**
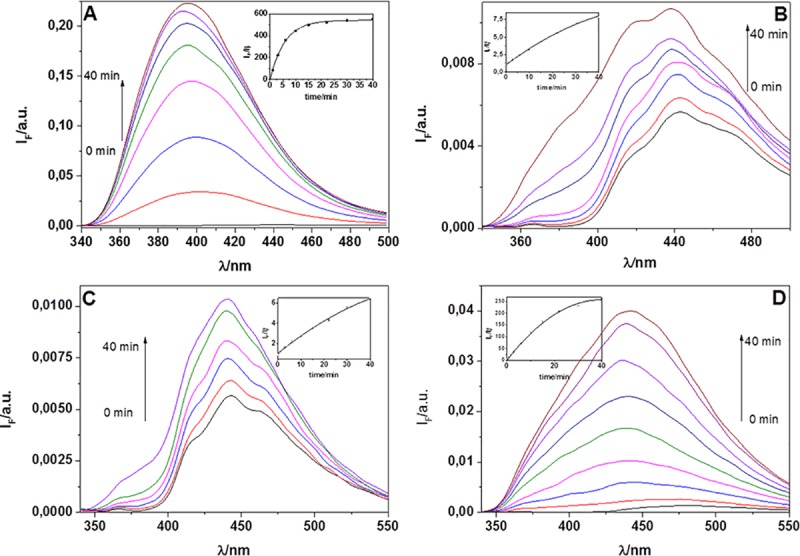
Fluorescence intensity changes for FN-6 (A), FN-7 (B), FN-8 (C) and FN-9 (D) upon reaction with ^1^O_2_ generated by irradiation of NMB in methanol. Inset: Fluorescence enhancement over irradiation time. Excitation wavelength was 330 nm.

The reaction of **FN-6** –**FN-9** with ^1^O_2_ produces likewise a notable increase on the fluorescence intensity, which is accompanied by a hypsochromic shift of the emission maxima (Fig 7). The rate of fluorescence growth is largest for **FN-6** and the fluorescence intensity of its oxidized form is >500-fold larger than that of the native form at the optimum excitation wavelength (330 nm).

The rate constants for overall ^1^O_2_ quenching by the dyads (*k*_q_) were determined by time-resolved luminescence spectroscopy (S3 Fig). Likewise, the reactive rate constants *k*_r_ were obtained by comparing the rate of consumption of the dyads to that of the reference compound dimethylanthracene. Both are collected in Table 4.

**Table 4:** Rate constants for overall (k_q_) and reactive (k_r_) quenching of singlet oxygen by furyl vinyl naphthoxazoles, singlet oxygen trapping efficiency (k_r_/k_q_), and quantum yields for singlet oxygen photosensitization.

	*k*_*q*_/10^7^ M^-1^ s^-1^		*k*_*r*_/10^7^ M^-1^ s^-1^	*k*_*r*_*/k*_*q*_	Ф_Δ_
	ACN	Methanol	Methanol	Methanol	Methanol
**FN-5**	1.2 ± 0.1	0.27 ± 0.03	0.22 ± 0.02	81%	0.003
**FN-6**	5.7 ± 0.4	10.4 ± 0.1	5.8 ± 0.1	56%	0.003
**FN-7**	0.18 ± 0.02	0.55 ± 0.06	0.25 ± 0.03	45%	0.009
**FN-8**	0.13 ± 0.01	0.16 ± 0.01	0.15 ± 0.02	94%	0.010
**FN-9**	1.2 ± 0.1	4.1 ± 0.3	1.8 ± 0.2	26%	0.004

Values of *k*_q_ in methanol included in Table 4 follow the order **FN-6** > **FN-9** >> **FN-7** > **FN-5** > **FN-8**. Thus, **FN-6** and to a lesser extent **FN-9**, are excellent ^1^O_2_ quenchers, with efficiency comparable to that of dimethylfuran (*k*_*q*_ = 2.4 x 10^7^ M^-1^ s^-1^) and methylfuran (*k*_*q*_ = 10.1 x 10^7^ M^-1^ s^-1^) in methanol [31]. Regarding the *k*_*r*_ values, some observations are worth highlighting: (i) Their reactivity with singlet oxygen follows the same trend as their overall quenching efficiency, **FN-6** and **FN-9** being the most reactive compounds; (ii) the trend is not affected by solvent polarity, as expected for a cycloaddition [4 + 2] reaction [50]; (iii) compared to the unsubstituted homologous **FN-5**, the methyl- and phenyl-substituted compounds **FN-6** and **FN-9** are 26- and 8-fold, respectively, more reactive; (iv) the least reactive compound is the Br-substituted **FN-8** as could possibly be expected by the electron-accepting character of the substituent, which decreases the electron density on the C-5 position of furan ring; (v) although it is not the compound with the highest singlet oxygen trapping efficiency (*k*_*r*_ / *k*_q_ = 56%), **FN-6** shows nevertheless the highest reactivity and therefore appears as the best candidate for being use as a singlet oxygen fluorescent probe.

### Structure-reactivity relationships

**Fig 8:**
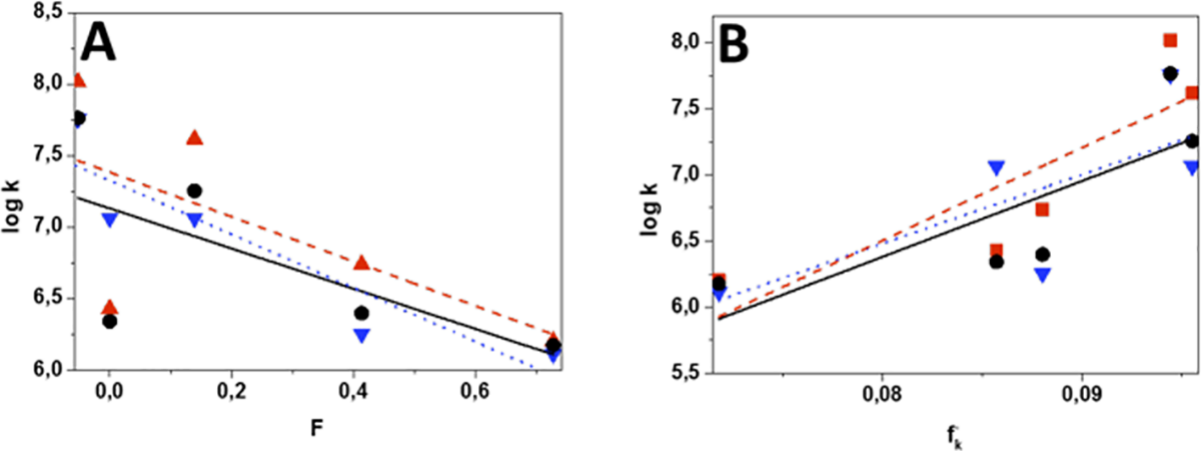
Linear correlation between the total rate constant for reaction of furyl vinyl naphthoxazoles and ^1^O_2_ with the F parameter of Swain and Lupton (A) and the Fukui condensed function for the electrophilic attack of ^1^O_2_ on the C-5 of furan ring (B). Solvents: ACN (black circles), methanol (red squares). Blue inverted triangles correspond to the reactive rate constant in methanol.

Substituents effects on the reactive rate constant were analyzed using the Hammett free energy relationship. Thus, the measured rate constants were correlated with σ_m_ and σ_p_ Hammett parameters [51] and with ***F*** and ***R***, the Swain and Lupton modified parameters [52], that redefine the substituent σ-parameter in terms of field effects, ***F*** (inductive and pure field) and the resonance effects, ***R***. Correlations of k_q_ with σ_m_, σ_p_ and ***R*** did not show clear trends. Better correlations in both methanol and ACN were obtained with the field parameter ***F***. Fig 8A shows that there is a good correlation between the inductive donor effect of the substituent and the dyads reactivity. Additional insight on the substituent effect upon the reaction rate and the electron density on the C-5 of the furan was obtained from correlations of the reaction rate with local Fukui coefficients. Condensed Fukui functions, **f**_**k**_^**+/-**^, account for physicochemical properties of atoms or functional groups in a molecule, such as the nucleophilicity and electrophilicity of different sites in molecule [53–55]. Fig 8B shows that there is a reasonable correlation between the values of the rate constants and the value of the Fukui condensed function for the C-5 position of the furan ring. In addition, the most reactive dyads, **FN-6**, with methyl and **FN-9**, with phenyl substituents, show the highest **f**_**k**_^**-**^ values and the lowest **F** values (low **F** values are associated with groups that have the greatest inductive effect).

**Fig 9:**
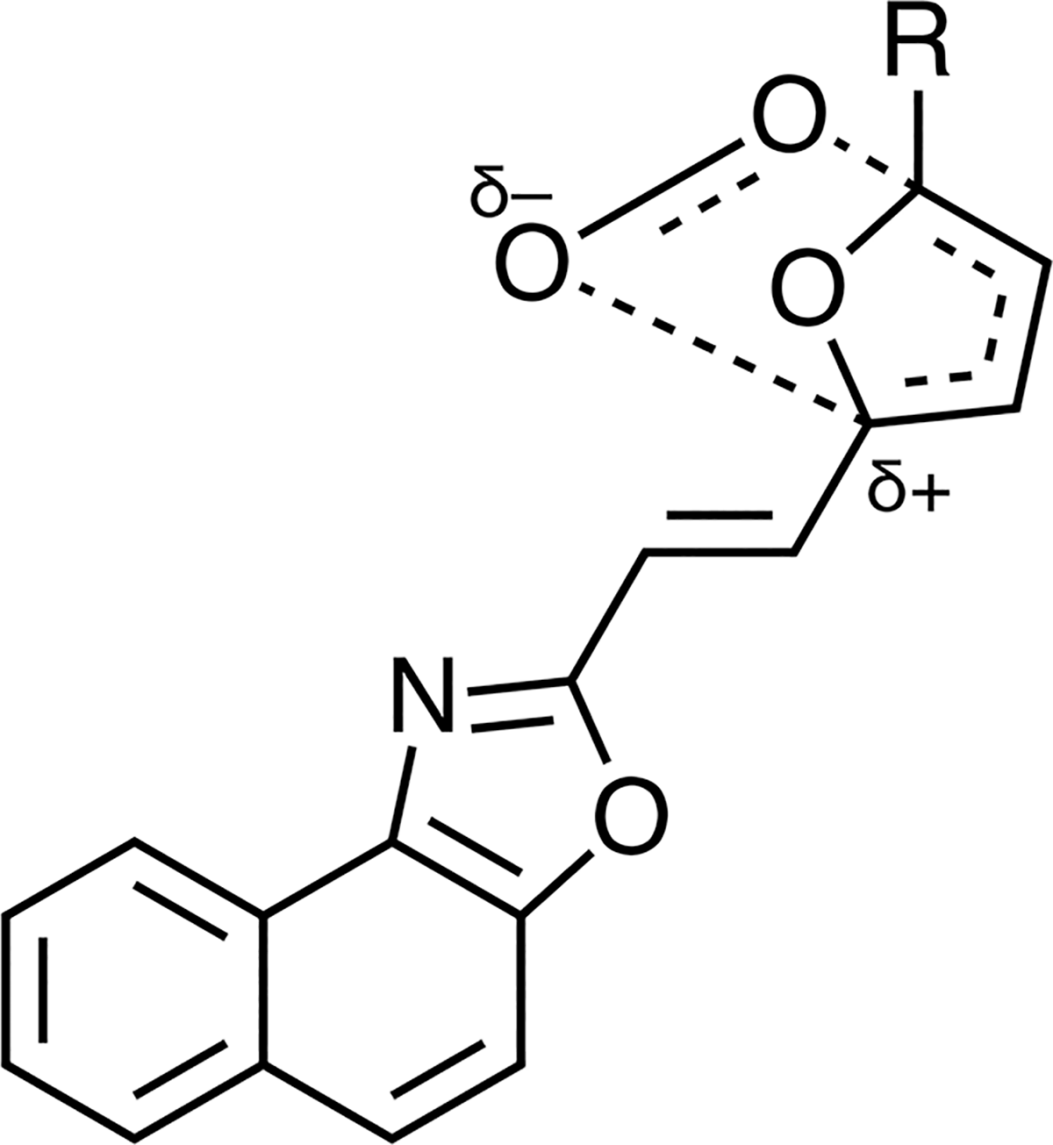
Exciplex structure for the partially concerted cycloaddition of ^1^O_2_ to furyl-substituted vinyl naphthoxazole.

These results suggest that the [4 + 2]-cycloaddition of ^1^O_2_ to the furan ring does not occur through a concerted mechanism, as for other furan derivatives [56], but possibly the attack proceeds in a partially concerted manner with a primary interaction of ^1^O_2_ with the center of greater electronic density, on the C-5 of the furan ring, as has been suggested by Lemp et al. in the cycloaddition of ^1^O_2_ to mono-substituted anthracenes [57], forming an exciplex of a "more open" structure with a large charge separation (Fig 9).

Moreover, mass spectra of main products obtained in the photooxidation of **FN-5** and **FN-6** (S4 Fig) shows the same fragmentation pattern, suggesting a common reaction mechanism. Both mass spectra are compatible with a classical photooxidation mechanism that involves a [4 + 2]-cycloaddition of ^1^O_2_ to the furan ring followed of methanolysis to give the final product (S5 Fig).

### Self-sensitization of ^1^O_2_ by the dyads and reactivity towards other ROS

A drawback of common ^1^O_2_ fluorescent probes is the evolution of fluorescence due to self-sensitization of ^1^O_2_ [58]. Other probes suffer from poor selectivity towards ^1^O_2_ and are capable of reacting also with other ROS. We investigated whether the naphthoxazole dyads are affected by the same problems. All dyads sensitized the production of ^1^O_2_ in methanol, however for **FN-6** and **FN-9** quantum yields (Ф_Δ_, Table 4) were 3-fold smaller than for the most popular probe SOSG [58]. Reactivity towards other ROS was also tested for the series **FN-5** –**FN-9**. We found negative results for all probes against superoxide (KO_2_) and H_2_O_2_ (S6 and S7 Figs). After 50 min. the extent of reaction is lower than 1,5% indicating a high degree of specificity for ^1^O_2_.

The results described in previous sections suggest that dyads which include a polycyclic aromatic naphthoxazole [1,2-d] system with a vinyl bridge at the 2-position of the heterocycle linked to a substituted furan ring with an alkyl group at carbon-5 are the ones with the highest fluorescent response. Accordingly, this structure could be employed to engineer a “click-on” probe to detect and quantify singlet oxygen in biological interest media, a potential solution if a quick response is needed in a test system. However, various characteristics of the biological systems must be considered to planning a successful probe: i) aromatic fluorescent molecules, such as naphthoxazole—furan dyads, could form complexes with proteins [59,60], altering the expected fluorescent increases after the ^1^O_2_ addition to the scavenger moiety and restricting substantively probe internalization in cells. Different approaches have been proposed to solve this problem, such as probe binding to nanoparticles, which maintains its reactivity towards ^1^O_2_, reducing the interaction with proteins [61–63]; ii) in biological media, a complex redox system maintaining cell viability is always present, which may affect the fluorescent response of the dyad if oxidant and/or reducing biomolecules react with the products yielded by the reaction between the dyad and ^1^O_2_. Nevertheless, various “off-on” fluorescent dyads based on polyaromatic systems have been employed to detect ^1^O_2_ in a diversity of cellular environments by the use of bioimaging techniques [59, 64–70]. Independent of the advantages or drawbacks of each probe, all of them afford highly repetitive results showing that, even in systems under oxidative stress, primary products of reaction with ^1^O_2_ (typically an endoperoxide), are stable under oxidizing or reducing conditions in cellular systems, a behavior also expected for furyl vinylnaphthoxazole endoperoxides; iii) the solubilization locus of the dyad can also modify the dyad fluorescent response due to the diversity of microenvironments in cells. However, we previously show that furan derivatives are very appropriate probes to monitor singlet oxygen dynamics in systems mimicking biological interest organizations [35,36]. In these systems, furan reactivity is nearly independent of the solubilization site although different sensitivities of the dyad towards ^1^O_2_ are expected, since the fluorescence quantum yield slightly increases in non-polar solvents; iv) at first, lasers employed to excite the dyad in cells after the reaction with ^1^O_2_ could involve high light intensities and/or focusing and it is reasonable to assume that high heat release could occur under these experimental conditions. These thermal effects open a new reaction pathway whereby thermally decomposition of endoperoxide could produce ^1^O_2_ and/or ^3^O_2_. Nevertheless, high molar absorptivity’s of dyads described herein can help to avoid severe thermal loads.

## Conclusions

Summing up, “click-on” dyads constructed by linking a furan ring to an aryloxazole at 2-position of the heterocycle via a vinyl bridge possesses appropriate properties to monitor and quantify singlet oxygen in solution. They show very low fluorescence quantum yields, are chemically stable, produce negligible amounts ^1^O_2_ by self-sensitization, and react selectively with ^1^O_2_ at high rate. Photooxidation yields a major product in which the structure of the fluorescent fragment is maintained but the non-radiative deactivation channel is cancelled upon oxidation, which leads to increasing the relative fluorescence up to an unprecedented 500-fold value. Reaction proceeds via a partially concerted [4 + 2] cycloaddition involving the formation of a loose or "open" structure exciplex, in which the oxygen is weakly bound to the furan ring. Dyads described herein are promising platforms to develop probes for monitoring singlet oxygen behavior in biological systems.

## Supporting information

S1 Fig HOMO orbitals for the dyads FN-1 (A), FN-4 (B) y FN-5 (C).
(TIF)

S2 Fig HPLC chromatograms of FN-5 before (solid line) and after (doted line) the photooxidation reaction using NMB as sensitizer in methanol (95% FN-5 consumption). (TIF)

S3 Fig Stern-Volmer plots for reactions of FN-7, FN-8 and FN-9 with ^1^O_2_ in metanol. Sensitizer NMB.
(TIF)

S4 Fig Low-resolution mass spectra of the main photooxidation product of FN-5 (A) and FN-6 (B) in methanol. Sensitizer: NMB.
(TIF)

S5 Fig Reaction mechanism of photooxidation of furyl vinyl naphthoxazoles. (TIF)

S6 Fig Reaction of FN-6 (A), FN-7 (B), FN-8 (C) and FN-9(D) with an excess of H_2_O_2_ in methanol. Reaction time 50 min.
(TIF)

S7 Fig Reaction of FN-6 (A), FN-7 (B), FN-8 (C) and FN-9(D) with an excess of KO_2_ in methanol. Reaction time 50 min.
(TIF)
