# Supplementary figures and images for: Structure-activity study of furyl aryloxazole fluorescent probes for the detection of singlet oxygen
Source: PLoS One. 2018 Jul 2;13(7):e0200006. doi: 10.1371/journal.pone.0200006 (PMC6028117; doi:10.1371/journal.pone.0200006)

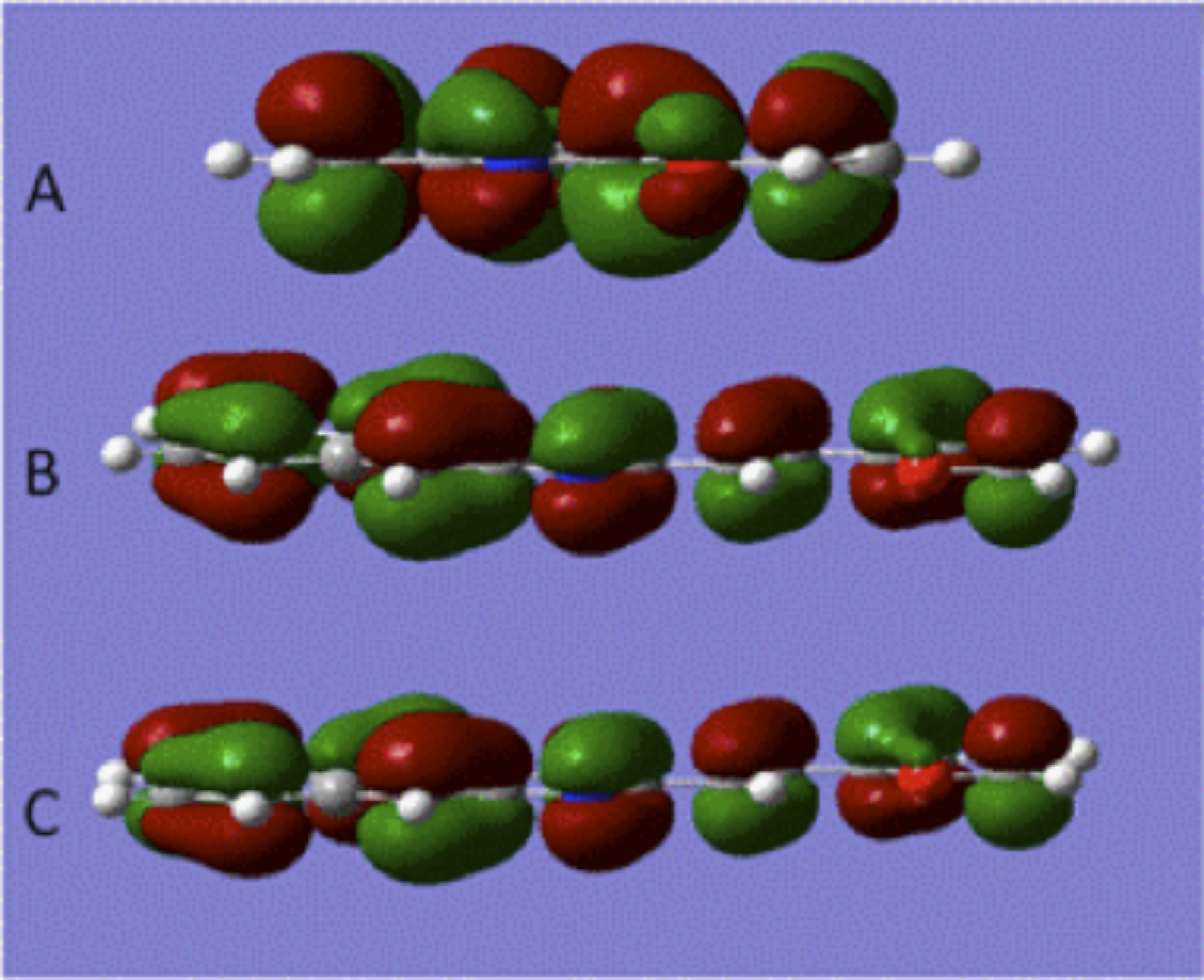

Supplement: S1 Fig — HOMO orbitals for the dyads FN-1 (A), FN-4 (B) y FN-5 (C). (TIF) [file pone.0200006.s001.tif]

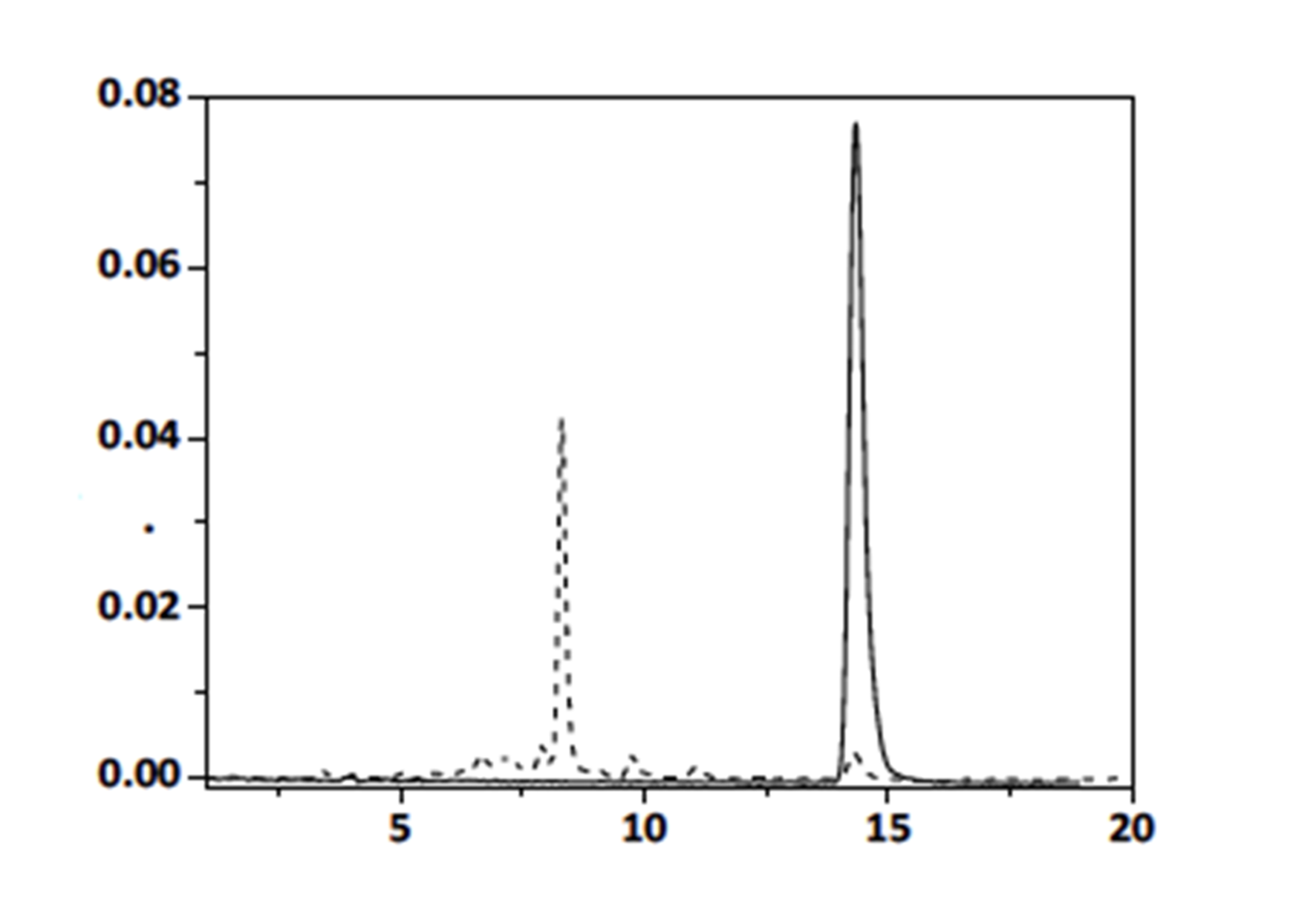

Supplement: S2 Fig — (TIF) [file pone.0200006.s002.tif]

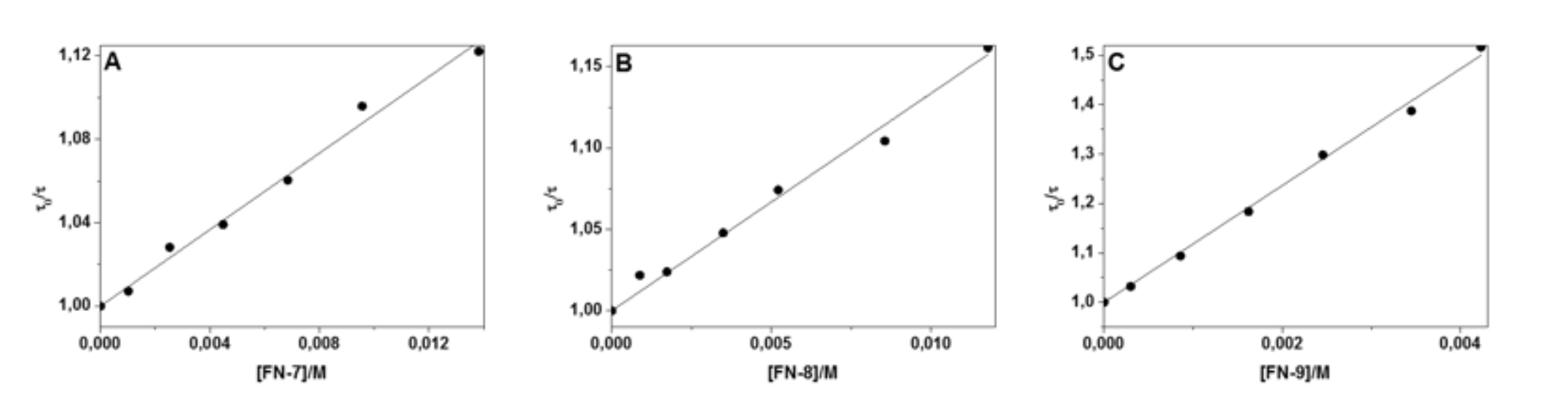

Supplement: S3 Fig — Sensitizer NMB. (TIF) [file pone.0200006.s003.tif]

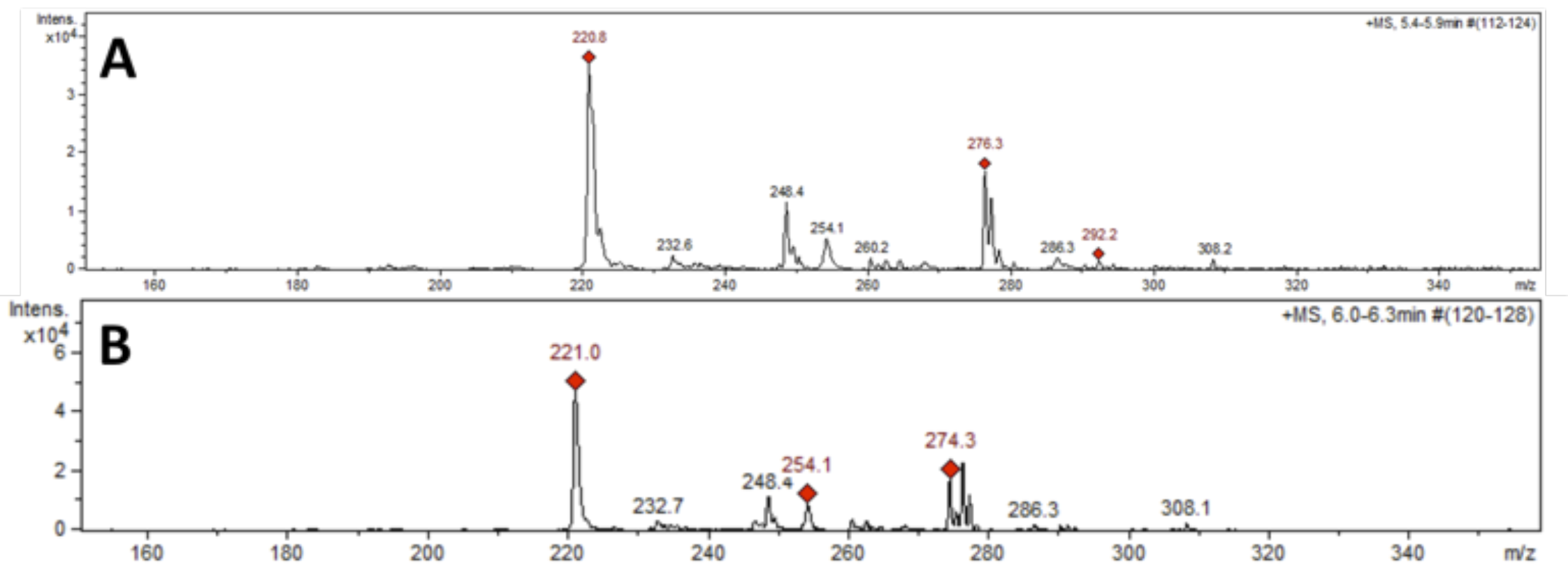

Supplement: S4 Fig — Low-resolution mass spectra of the main photooxidation product of FN-5 (A) and FN-6 (B) in methanol. Sensitizer: NMB. (TIF) [file pone.0200006.s004.tif]

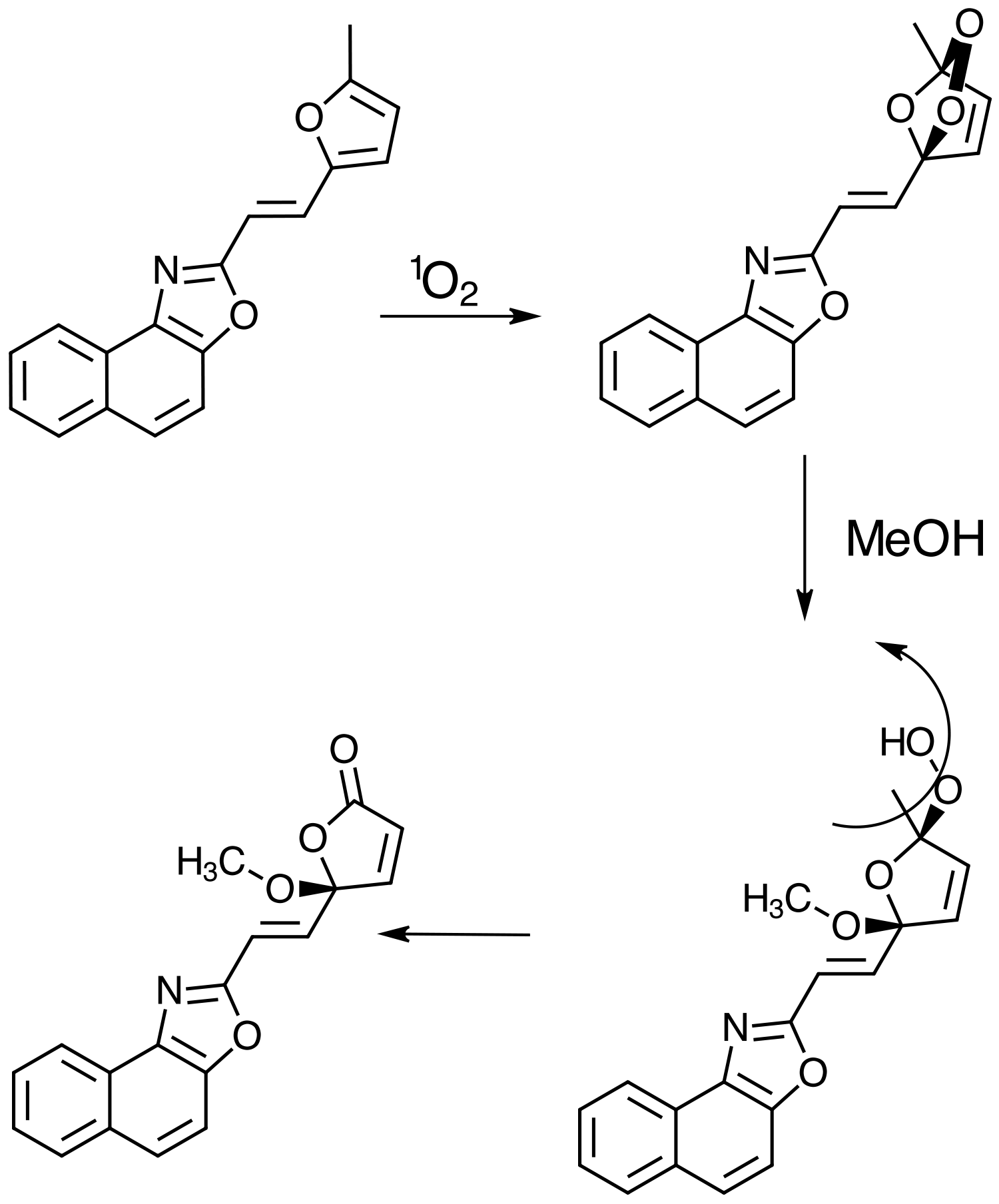

Supplement: S5 Fig — (TIF) [file pone.0200006.s005.tif]

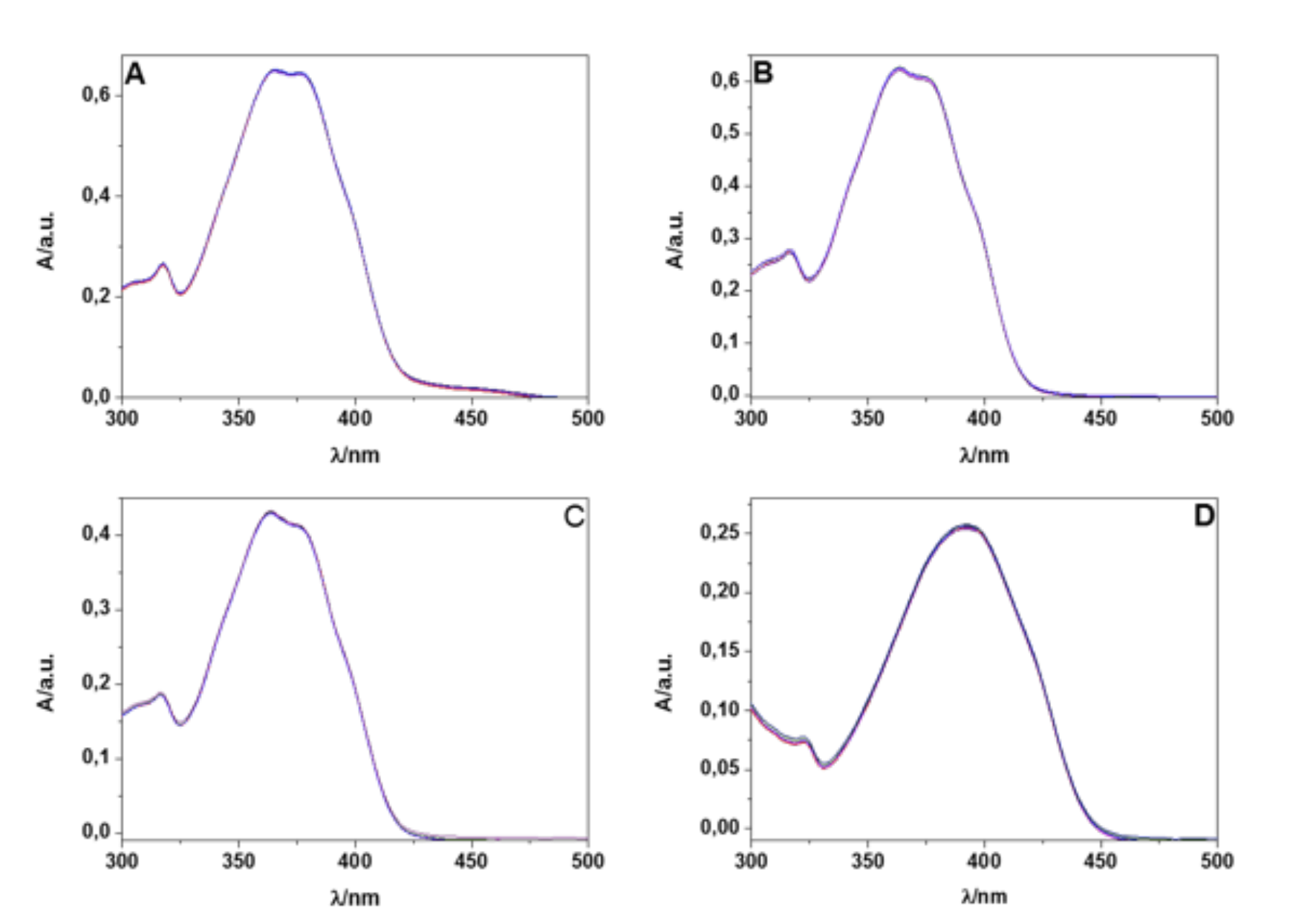

Supplement: S6 Fig — Reaction of FN-6 (A), FN-7 (B), FN-8 (C) and FN-9(D) with an excess of H2O2 in methanol. Reaction time 50 min. (TIF) [file pone.0200006.s006.tif]

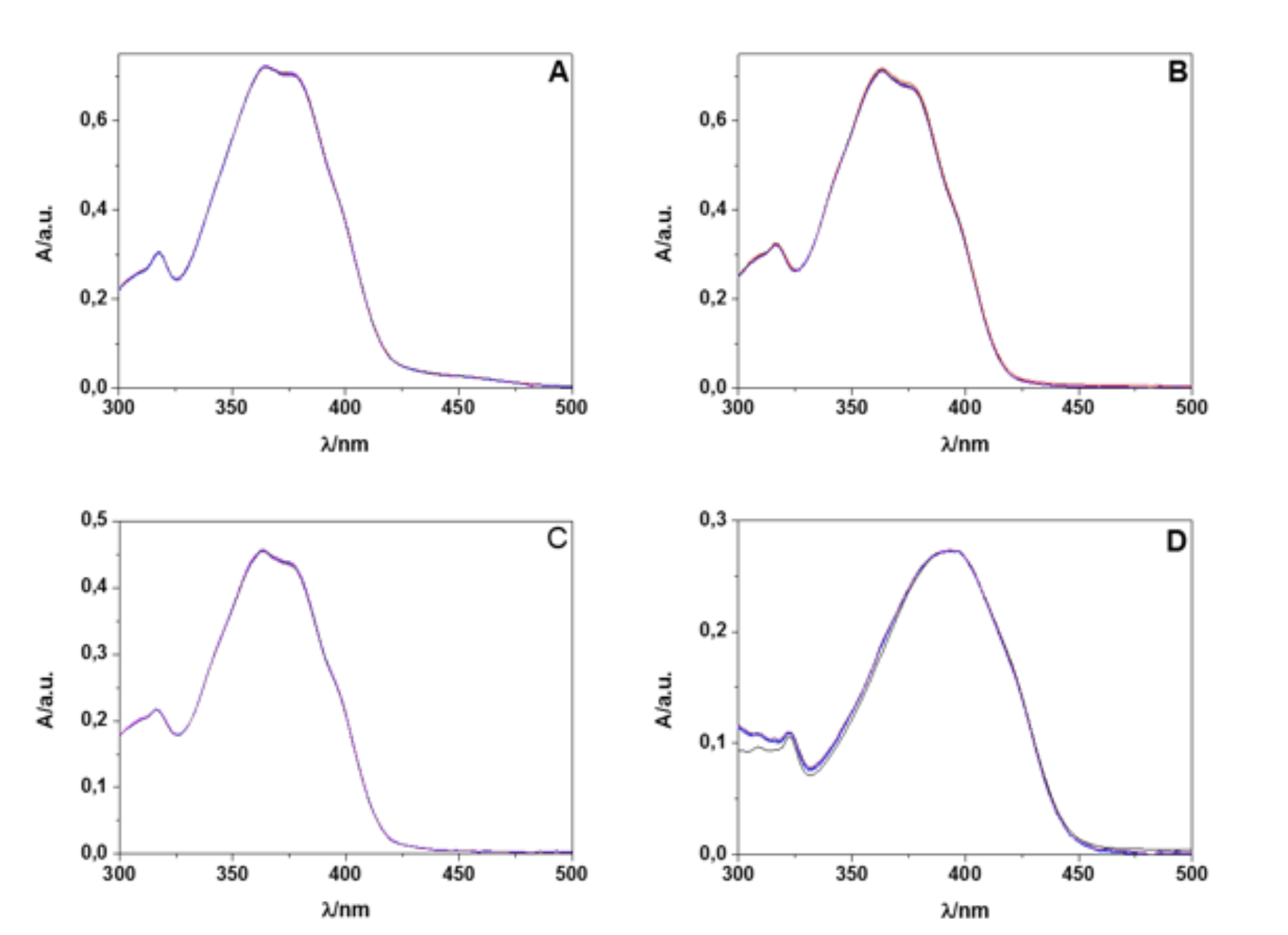

Supplement: S7 Fig — Reaction of FN-6 (A), FN-7 (B), FN-8 (C) and FN-9(D) with an excess of KO2 in methanol. Reaction time 50 min. (TIF) [file pone.0200006.s007.tif]
